# Intergroup conflict: origins, dynamics and consequences across taxa

**DOI:** 10.1098/rstb.2021.0134

**Published:** 2022-05-23

**Authors:** Carsten K. W. De Dreu, Zegni Triki

**Affiliations:** ^1^ Institute of Psychology, Leiden University, Leiden, The Netherlands; ^2^ Center for Research in Experimental Economics and Political Decision Making, University of Amsterdam, Amsterdam, The Netherlands; ^3^ Department of Zoology, Stockholm University, Stockholm, Sweden

**Keywords:** social species, intergroup conflict, contests, cooperation, fitness, natural selection

## Abstract

Although uniquely destructive and wasteful, intergroup conflict and warfare are not confined to humans. They are seen across a range of group-living species, from social insects, fishes and birds to mammals, including nonhuman primates. With its unique collection of theory, research and review contributions from biology, anthropology and economics, this theme issue provides novel insights into intergroup conflict across taxa. Here, we introduce and organize this theme issue on the origins and consequences of intergroup conflict. We provide a coherent framework by modelling intergroup conflicts as multi-level games of strategy in which individuals within groups cooperate to compete with (individuals in) other groups for scarce resources, such as territory, food, mating opportunities, power and influence. Within this framework, we identify cross-species mechanisms and consequences of (participating in) intergroup conflict. We conclude by highlighting crosscutting innovations in the study of intergroup conflict set forth by individual contributions. These include, among others, insights on how within-group heterogeneities and leadership relate to group conflict, how intergroup conflict shapes social organization and how climate change and environmental degradation transition intergroup relations from peaceful coexistence to violent conflict.

This article is part of the theme issue ‘Intergroup conflict across taxa’.

## 1. Introduction

**Figure 1.**
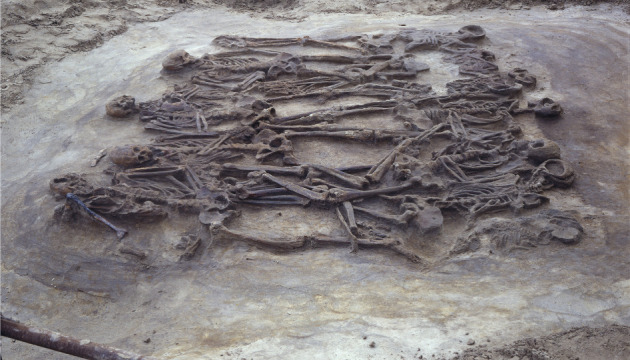
Violent intergroup conflict in humans. The burial at Wassenaar contains the remains of 12 people possibly murdered during an out-group raid on their settlement (approx. 3400 BP; reproduced with courtesy of Dr Louwe Kooijmans and Faculty of Archaeology, Leiden University).

The members of the Historical Society of Wassenaar, a small coastal town in the Netherlands, knew their region had been dotted with human settlements for millennia. As amateur archaeologists, they had regularly found the remnants of pottery and jewellery that could be dated back thousands of years. Yet in April 1987, they made a unique discovery—a Bronze Age burial site with 12 well-preserved skeletons of men, women and children (figure 1). The 12 belonged to several families and were buried all at the same time, around 3400 BP. Each skeleton had clear marks of lethal violence—the 12 were most likely killed when a neighbouring group raided their settlement [1].

The excavation at Wassenaar changed the then prevailing view of the Bronze Age as an Era of Peace [2–4]. Extant work in evolutionary archaeology and anthropology strongly suggests that intergroup conflict has been a constant throughout human history [5–11] (but see [12]). Moreover, and in parallel, it has become clear that intergroup conflict and warfare^1^ are far from unique to the human species. For example, nonhuman primates, including chimpanzees (*Pan troglodytes*) and baboons (e.g. *Papio cynocephalus*), engage in violent conflict with neighbouring groups [13–18]. Intergroup conflict has also been observed in spider monkeys (*Ateles paniscus*) [19], hyaenas (*Crocuta crocuta*) [20], wolves (*Canis lupus*) [21], meerkats (*Suricata suricatta*) [22], banded mongooses (*Mungos mungo*) [23,24], in various group-living birds [25–27] and social fishes [28]. Social insects raid neighbouring colonies and kill enemy conspecifics [29–33].

Exceptions aside, intergroup conflict can be exceedingly costly to the involved individuals, their groups and the population at large. In humans, political revolts, civil wars and interstate conflict since 1946 resulted in over 40 million people killed [34,35]. Chimpanzees kill and die from battle-related injuries [16], and inter-colony warfare among social insects such as ants and bees can kill tens of thousands [31,36]. Moreover, and in addition to battle-related trauma, intergroup conflict has, across species, been linked to environmental degradation and famine, migration and forced relocation and the spreading of infectious diseases [26,37–39].

These two observations—intergroup conflict is seen across taxa and can be exceedingly costly—raise fundamental questions about the origins, dynamics and consequences of intergroup conflict. For example, we can ask when and where intergroup conflicts emerge and why: are there cross-taxa commonalities in the preconditions for intergroup conflict? Relatedly, we can ask what (groups of) individuals gain from initiating and escalating conflict with neighbouring groups of conspecifics: what pay-offs make the opportunity costs and risk of (sub-) lethal injury worthwhile to pursue? Finally, we can ask whether and how recurrent intergroup conflicts shape the social organization of groups (e.g. group size, group composition) and, as a consequence, population structures.

This theme issue addresses these and related questions. It combines state-of-the-art theory and reviews alongside new research on intergroup conflict from the perspectives of neurobiology, animal behaviour, anthropology, economics and evolutionary theory. It provides, for the first time, to our knowledge, a side-by-side treatment of intergroup conflict across taxa, detailing the origins and consequences of intergroup conflict. Each in their own way, the contributions provide insight on intergroup conflict for social insects [32,33], social fishes [40], group-living birds [26,27], non-primate mammals such as banded mongoose [24], lions and wolves [17], several monkey species [41–43], chimpanzees [18,44] and humans [45–47].

**Figure 2.**
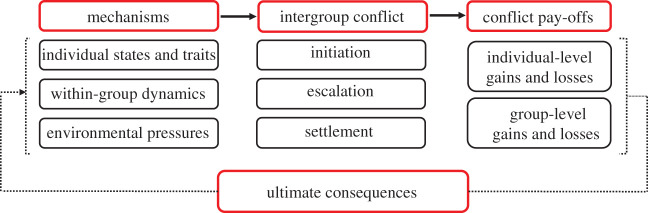
Framework for analysing intergroup conflict. Mechanisms at the individual, group and environmental level of analysis producing the initiation, escalation and settlement of intergroup conflict, with conflict pay-offs for individuals and their groups. Recurrent intergroup conflict within and across species' generations has possible ultimate consequences on the individual, group and environmental level of analysis. (Online version in colour.)

Here, we introduce the theme issue and its contributions with a coherent framework of intergroup conflict (figure 2). In §2, we model intergroup conflict as a multi-level ‘game of strategy’ in which individual animals cooperate in groups to compete against outsiders [48–52]. In §3, we identify general, cross-species mechanisms for the initiation and escalation of intergroup conflict. In §4, we review the possible consequences of intergroup conflict. We distinguish between immediate conflict pay-offs and other, more indirect proximate consequences of intergroup conflict that occur within the lifetime of (groups of) individuals and possible ultimate consequences of recurrent and impactful intergroup conflict across generations and evolutionary timescales.^2^ Section 5 concludes with innovative advances on our understanding of intergroup conflict that emerge from the contributions to this theme issue.

## 2. The anatomy of intergroup conflict

Many species across taxa require access to territories and resources that are shared with and also demanded by other species, conspecifics included. This can create competition and conflict both within and between groups of individuals [59]. Indeed, for intergroup competition to emerge, it is quintessential that individuals are nested in groups and that groups are nested in a structured population of multiple groups (see e.g. [60–62]). Groups can differ in size and in internal cohesiveness, and group boundaries can be permeable such that individual members are more or less able to migrate into adjacent groups or form new groups. Regardless, groups within such structured populations may collectively compete over scarce resources such as territory and shelter, access to food and water and mating opportunities [48–50,52,63]. In humans, intergroup competition and conflict sometimes also involve immaterial resources like political influence, the truth and the validity of worldviews [64,65].

During intergroup conflict, individuals from opposite sides may fight in one-to-one combat or, alternatively, contribute metabolic energy, skills, insights and material resources to a collective ‘fighting capacity’ like an army or building a collective defence system. In either case, the conflict can be about something both groups desire, yet only one group can have. An example is that of two ant colonies aiming to feed on a resource that provides enough food for only one colony [36]. At other times, conflict is about something that one group wants and the other group owns and defends [53,66,67]. An example is that of a raiding party invading its neighbour's territory to capture their livestock [68]. Border patrols in chimpanzees are another example, where one group uses violence against intruders to protect its territory [16,18,44].

As these examples show, intergroup conflict can be modelled as a two-level ‘game of strategy’ in which (i) individuals within groups cooperate at a personal cost to (ii) generate a group-level ‘fighting capacity’ to compete against (groups of) outsiders. Accordingly, groups need to ensure that (enough of) their members participate—groups need to overcome *problems of cooperation* that emerge from individual temptations to free-ride on others' cooperative efforts and avoid the opportunity costs and injuries associated with joining intergroup conflict [69]. Relatedly, groups sometimes face problems of coordination [70–72], for example, when they need to calibrate how much effort each individual expends [50,67] or when they need to decide when to intrude into enemy territory and when to ‘lay low’ [68,73] (further see §3b).

All else equal, the group that can raise more contributions to conflict is most likely to settle the conflict in its favour or prevent being defeated. This happens, for example, when one group is substantially larger than its competitor [74] or when the group solve its collective action problems better than its rivals [69,75]. In these and similar cases, intergroup conflicts can take the form of ‘*win–lose’ conflicts*—both sides waste efforts but individuals in victorious groups typically earn a share of the resources captured, while defeated groups remain empty-handed or worse. However, not all intergroup conflicts result in win–lose outcomes. In many cases, for example, when rivalling groups are of equal strength and invest equally in conflict, conflict settlement can take the form of a ‘*lose*–*lose’ conflict*. In fact, game-theoretic analyses and empirical observations of intergroup conflicts across social vertebrates reveal that in the majority of cases, group defence is sufficiently strong to prevent defeat [73] (also see [76,77]). Yet both failed attacks and successful defences are energetically costly and can involve physical trauma and casualties—neither side wins, and both lose. Finally, and at least in humans, conflict sometimes ends in a mutually beneficial exchange on interests and positions, akin to a ‘*win*–*win’ conflict* in which both sides earn some share of the contested resource that at least partially offsets the costs of participation in conflict [78,79].

That often both groups survive intergroup conflict means that individuals on both sides can recover and reproduce. It also means that rivalling groups can adapt to each other, developing strategies for preventing or more successfully winning future contests and competitions [76,80–82] (also see [83]). As we will discuss in §§4 and 5, both (not) winning and (not) losing impact individual and group survival and fitness and can shape the social organization of groups and populations.

## 3. Initiating and escalating intergroup conflict

In theory, the mechanisms that produce conflict are entwined with the pay-offs of recurrent intergroup conflict. Accordingly, one way to understand when and how individuals and groups initiate and escalate intergroup conflict is to ask about the expected value or ‘current utility’, of contributing to conflict (*viz.* [84]). In addition, we may identify the control mechanisms that groups use to solve emerging problems of cooperation and coordination during intergroup conflict and how changes in the groups' environment modulate group members’ engagement in out-group aggression.

### (a) Individual participation in group conflict

Borrowing from standard economic theory, we assume that choosing a conflictual path of action becomes more attractive for individuals, relative to less conflictual options, with increases in several characteristics of the decision situation [51,52] (also see [84,85]). Adding to past literature, several contributions to this theme issue converge on five key arguments that determine what we can call the ‘conflict participation function’ *p* for individual *i*:

3.1 $$p_{i}=f(v,\;\gamma ,\;\alpha_{I},\;\alpha_{O},\;\beta ),$$

where *v* refers to the expected material benefits of participating, such as additional food or territorial access; *γ* captures the expected benefits of participating in terms of gains in status and reputation among the members of one's own group (‘in-group); and *α*_I_ and *α*_O_ denote the value the individual attaches to the welfare and well-being of (members of) their in-group and rivalling out-group, respectively; *β* denotes out-group threat as the magnitude of expected out-group aggression. Accordingly, arguments *v* and *γ* capture the individual's ‘selfish’ motivation, and *α*_I_, *α*_O_ and *β* capture the individual's ‘social’ motivation to join intergroup conflict.

Each argument in *f* may have a *positive* or *negative* effect on the probability of participating in group conflict. Accordingly, we can expect participation when, first, the expected material benefits of participating increase (∂*p*_i_/∂*v* > 0). Being part of a winning group promises potential material benefits such as a share in conquered resources and/or mating opportunities (*viz.* spoils of war). Mathew [46], for example, shows how the promise of adding cattle to one's household can further motivate members of the Turkana, a tribal society in East Africa, to join raids on adjacent communities. Sometimes, key group members, such as group leaders, may reap larger benefits than the rest of the group members or are at relatively low risk during the fighting [86,87]. Sankey *et al*. [24] show evidence for this in banded mongooses where ‘exploitative’ leaders initiate group conflict and benefit disproportionately relative to the rest of the group. In addition to these material benefits, conflict participation may increase status-ranking (∂*p*_i_/∂*γ* > 0) (i.e. costly signalling [88,89]). Status and reputation can, in turn, provide access to valuable resources such as mating opportunities and food [15,43,90].

In addition to the (im-) material benefits to the individual themselves, participation in conflict also depends on the individual's social preferences—the value attached to the well-being of in-group members (*α*_I_) and out-group members (*α*_O_) [51]. From both inclusive fitness theory [91] and work on social preferences in (non-)human primates [92,93], we can assume that individuals care about the welfare and well-being of genetically related or culturally similar others [49,93]. Such *α*_I_ affords parochial cooperation—energetically costly actions that support the survival and reproductive success of conspecifics within one's group [91] (also see [94,95]. When winning group conflict benefits in-group members, parochial in-group concern can motivate conflict participation (∂*p*_i_/∂*α*_I_ > 0) [96–99]. As a case in point, Triki *et al*. [40] review evidence that oxytocin, a neuropeptide that is structurally preserved across taxa, mediates social affiliation and care and links to participation in group conflict for a range of vertebrates, including social fishes, gregarious birds and various mammals, including humans.

Rusch [51] provides a succinct review of social preference models developed in economics that further detail how social preferences underlie conflict participation. Key in these models is that social preferences differ for in-group and out-group members, respectively. Typically, group conflict has a negative impact on (members of) the rivalling out-group. Individuals may be averse to such negative impacts and have pro-social concerns for out-group conspecifics (∂*p*_i_/∂*α*_O_ < 0). This should *reduce* conflict participation and afford cooperative intergroup interactions as seen in, for instance, ant species [33], bonobos [18,100] and humans [101–103]. However, in the case of antisocial preferences for out-groups, individuals may initiate and escalate conflict out of ‘spite’ [104–106]. Revenge killing in primates [13] may reflect such negative concern for out-group conspecifics. Thus, having negative concern for out-group conspecifics may suffice to produce conflict participation [105,107].

The final argument in equation (3.1) that modulates conflict participation is the magnitude of out-group threat *β*. Genetic and cultural relatedness alongside shared histories can create differential beliefs among individuals about the extent to which in-group versus out-group conspecifics cooperate or fight. Individuals contribute to group defence when they expect (members of) out-groups to initiate group conflict (∂*p*_i_/∂*β* > 0) [23,97,108,109]. Indeed, humans strike pre-emptively to neutralize the threat from out-groups [110,111], primates such as verreaux's sifakas (*Propithecus verreauxi*) are more likely to join intergroup conflict the more actively participating individuals are in the out-group [112], and meerkats perform energetically costly ‘on-guard’ behaviours to warn their group mates against predators and enemy conspecifics [113].

Whereas each argument in equation (3.1) may contribute to conflict participation, effects need not be linear. For example, material benefits (argument *v*) may have a decreasing marginal return, with more of the same benefit being increasingly less ‘motivating’. Likewise, some magnitude of out-group threat *β* may lead to conflict participating, but too high magnitude may lead to fleeing or surrender, rather than fighting. Second, some arguments in the conflict participation function may be present in some animal species and absent in others. In some species, conflict participation is mainly ‘opportunistic’ and grounded in *v* and *γ*. In many species, however, social preferences (elements *α*_I_, *α*_O_) have been identified as contributing factors as well. Third, arguments may reinforce or counteract each other and differ in relative contribution. For example, in-group concerns *α*_I_ may be strong enough for the individual to join, despite the expected personal benefits (*v, γ*) being low or even negative.

### (b) Coordinating collective action

The conflict participation function specified in the previous section leaves open the possibility that some group members may be driven by different arguments than others and that there are within-group heterogeneities in the degree to which group members participate in intergroup conflict. Such within-group heterogeneities in the expected value from conflict participation can lead some individuals to initiate conflict where other group members would not, and they can create or aggravate problems of cooperation and coordination that can threaten success in intergroup competition and conflict. For example, when ‘spoils of war’ are distributed among group members regardless of how much individuals contributed to fighting, individuals may withhold their contributions and ‘lay low’. Such free-riding weakens the likelihood the group wins and may leave all group members worse off.

Groups solve problems of cooperation by influencing the various arguments in the individual conflict participation function. In humans, for instance, groups shape the rules of distribution, such that some members can expect a larger share in the spoils than others (*v* in equation (3.1)). Mathew [46] shows how members of the Turkana, a tribal society in East Africa, build coalitions by promising ‘reluctant’ community members an enticing share in the spoils of war, and Bshary *et al*. [43] discuss how in vervet monkeys, females incentivize males to engage in intergroup conflict with mating opportunities. Relatedly, groups can solve the problem of cooperation by rewarding bravery with status and reputation (*γ* in equation (3.1)) [114,115]. Conversely, groups can sanction free-riders through peer punishment, effectively reducing the individual's social and material benefits from free-riding, something seen in various mammalian species [62,116,117], in social fishes [118] and insect societies [119]. At least in humans, punishing members who did not fight increases their conflict participation [68,120,121].

Sharing rules, punishment and rewards alter the ‘selfish’ benefits from participation in group conflict. Groups also increase participation by acting on their group members' social preferences. In humans, leader rhetoric sometimes aims at creating hatred for rivalling out-groups (*α*_O_) [46,62], and social bonding rituals increase parochial in-group concern (*α*_I_) [122–124]. Lemoine *et al*. [18] review evidence that such a mechanism generalizes beyond humans—collective grooming and food sharing in chimpanzees prior to intergroup encounters can increase social ties among group members.

To solve problems of coordination—*who* contributes *what* and *when*—individuals within groups can specialize in some tasks and not others. Task specialization can be horizontal, dividing the group into ‘fighters’ and ‘producers’ [57]. Such division of labour enables individuals to ‘heuristically’ decide whether to join and expend effort on group conflict or not. Individuals can also take turns in which role they assume. In a range of group-living mammals, male and female members take distinctly different roles, with males often being more directly involved in coalitional fighting [17]. Or to give another example, meerkats take turns in standing ‘on guard’ [113,125], and humans take shifts in positioning themselves at the back or frontline of group fighting [126]. In addition, solutions to the problem of cooperation—sanctioning free-riding, rewarding bravery, and strengthening social bonds—can be selectively applied to ‘fighters’ but not to ‘producers’.

Task specialization can also be vertical, carving up the group into leaders and followers (see [17,24]). Vertical specialization centralizes decision-making and can make collective action more efficient and effective [127–129]. For example, collective movement in African wild dogs is predicted by a few dominant individuals taking the initiative [130], and a pack of hunting wolves closing in on large prey awaits the initiative of its most senior member to withdraw or attack [17,131]. In human groups, ‘leading-by-example’ is an effective means to coordinate collective action [69,132,133] and can make out-group attacks more effective [73,134,135].

In theory, horizontal and vertical task specializations can emerge independently of each other—sub-groups of fighters and producers can each have (or not) a leader. Furthermore, both horizontal and vertical specializations can be ‘hard-wired’. For example, in social insects such as ants and bees, the individual's role and hierarchical position have a strong epigenetic element [136,137], and in many social vertebrates, task specializations are sexually dimorphic with males and females taking up different roles and hierarchical positions during intergroup conflict [17,43]. Sometimes, however, task specializations are flexible and change over time. Human groups deliberately select some community members to join raids on neighbouring tribes, with partner selection depending on both individual abilities and reputations [46,63,114,138]. Such skill-based partner choice to coordinate collective action is seen in other species too, including chimpanzees [139], gorillas (*Gorilla g. gorilla*) [140], marmosets (*Callithrix jacchus*) [141], harbour porpoises (*Phocoena phocoena*) [142] and groupers (*Plectropomus pessuliferus marisrubri*) [143].

### (c) Environmental pressures

For many group-living species, intergroup conflicts emerge over scarce resources. By implication, changes in the availability of resources such as food and territory can impact whether and where intergroup conflicts emerge. Resource scarcities can increase owing to exogenous events that groups and individuals poorly control. For example, climate change, alongside extreme flooding and droughts, can degrade the natural environment and make local food supply problematic. Population-level resource scarcity can also increase because of endogenous conditions, like growth in group sizes [144]. With high reproduction and low mortality, prospering groups increase in size and hence need larger territories with access to more food, water and shelter. By cooperating well, groups can thus endogenously create their own ‘carrying-capacity stress’ [49,145,146].

Carrying-capacity stress increases the probability of between-group competition and conflict [147–152]. First, carrying-capacity stress can bind individuals within groups and promote parochial other-concern (*α*_I_ in equation (3.1); see [153]). De Dreu *et al*. [45] provide experimental evidence that environmental unpredictability and ensuing carrying-capacity stress lead to parochial in-group concerns and more out-group aggression. Their findings resonate with findings on clan formation in spotted hyaenas [154] and can explain how climate change and economic shocks link to political revolts and civil conflicts in humans [155–157]. Second, carrying-capacity stress increases the expected benefits from invading adjacent territories. The increased risk of starvation makes the risk of lethal injury from fighting comparatively low and aggressing out-groups more ‘attractive’ (element *v* in equation (3.1)) [45,158]. Gareta García *et al*. [41] provide data conducive to this possibility where groups of vervet monkeys are more likely to meet and compete with others in areas with higher ecological value. Likewise, Brown *et al*. [42] find that victorious groups of red-tailed monkeys in Kibale National Park, Uganda, experience greater access to and consumption of food than defeated groups, yet this largely recoups earlier food deficits. Hunger drives groups into intergroup contests, and the hungrier a group is, the more likely it is to win a conflict.

## 4. Consequences of intergroup conflict

Intergroup conflict can have a range of consequences that include material and reputation gains and losses, physical trauma and exhaustion, physiological stress and proneness to disease. These and other consequences may be distinctly different for victorious compared to defeated groups, and they can apply to individuals, their groups and entire populations. Furthermore, consequences of intergroup conflict can be separated into immediate and proximate effects on ecological timescales, on the one hand, and distal and ultimate consequences on evolutionary timescales, on the other hand (also see [84,85]). For immediate consequences that occur within the lifetime of individuals, the critical question is how conflict dynamics, and the way conflict is resolved, affects the involved individuals and their groups during (parts of their) life. For distal and ultimate consequences, the critical question is whether and how intergroup conflict and its consequences shape, over generations and evolution, the biological and perhaps cultural development of (groups of) individuals. For example, bacteria and microbes have a wide range of chemical, mechanical and biological weapons to defend against and kill competitors that may have evolved through recurrent inter-strain warfare [159,160].

### (a) Pay-offs and proximate consequences

Conflict pay-offs for (groups of) individuals involved in the conflict are closely entwined with the elements of participation in a conflict (equation (3.1)) [26,49]. Intergroup conflict impacts the individual's food and territorial resources, social status and mating opportunities [24,43,114,115]. These impacts can be distinctly different for some group members compared to others. In some species, males are more likely to be directly involved in group conflict than females, and the impact of conflict differs significantly for male and female group members [17]. Moreover, Morris-Drake *et al*. [26] discuss a range of individual-level pay-offs, some being more ‘hidden’ than others. For example, non-participating individuals (e.g. ‘producers’; see §3*b*) may benefit from participating group members being injured or killed during conflict—they can usurp their vacated nest-sites, mating partners or leadership positions. Being part of a losing group may provide some individuals with perhaps unanticipated positive pay-offs.

Morris-Drake *et al.* [26] highlight that, in addition to the individual and group-level pay-offs, intergroup conflict can have a range of indirect consequences. For example, the physical contact among members of rivalling groups can facilitate the spreading of infectious diseases and individuals not involved in fighting may endure significant suffering—in humans, for example, each year about one million children younger than 5 years of age die because of conflict-related neglect and malnutrition [161]. Conversely, experiments with human participants reveal that individual exposure to war violence increases parochial cooperation and, at the local level, group cohesion and social bonding [123,162]. Likewise, allopreening frequency increases in groups of the green wood hoopoe following an intergroup conflict (*Phoeniculus purpureus*) [163], and chimpanzees engage in more cooperative grooming after aggressive intergroup encounters [18,164]. These social behaviours can reduce the presence of ectoparasites, alleviate stress from conflict interactions [165,166] and relax within-group competition and aggression. The proximate consequences of intergroup conflict can thus both reduce and increase individual and group welfare and well-being.

### (b) Distal and ultimate consequences

Some pay-offs from engaging in intergroup conflict, such as increased access to mates and resources, can lead (some individuals within) groups to reproduce comparatively better than others. Through differential survival and reproduction over time and generations, intergroup conflict may have ultimate consequences for (groups of) individuals [167].

**Figure 3.**
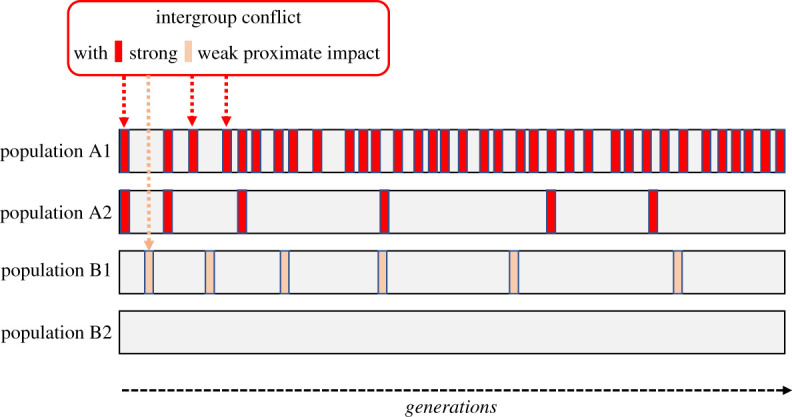
Intergroup conflict as a mechanism in the evolution of group-living species. Populations or species may face intergroup conflict or not within and across generations (e.g. comparing populations A1, A2 and B1 to population B2), and when intergroup conflict occurs, it may be more or less frequent (e.g. comparing populations A1 and A2) and with strong versus weak proximate consequences (e.g. comparing populations A2 and B1). Populations can refer to an entire species or to a species with lineages living in distinct ecological niches. The frequency and impact of intergroup conflict within and across generations shape ultimate consequences in terms of survival and fitness and how groups are socially organized in terms of size, cohesiveness and horizontal and vertical task specializations. (Online version in colour.)

Asking about the possible distal and ultimate consequences of intergroup conflict shifts the level of explanation from considering intergroup conflict as the result of individual and group-level mechanisms, as discussed in §3, to intergroup conflict as a mechanism or evolutionary selection pressure (figure 3) [12]. How this works can be difficult to assess, especially for species with extended lifespans. Archival and historical analyses can help to identify how over time intergroup conflict relates to reproductive success and group and population sizes (e.g. [44,168]). Comparative phylogenetic analysis of species with recurrent intergroup conflict can shed light on how conflict shapes (sub-populations of) group-living species (see [17,43]). Last but not least, evolutionary agent-based models and simulations (e.g. [61,169,170] can be useful to compare the evolution of traits in structured populations with and without recurrent intergroup conflict).

For intergroup conflict to have ultimate consequences, it needs to occur with some frequency in an individual's life, and it needs to be impactful (figure 3) [163,171]. Among group-living birds, for example, intergroup conflict often occurs yet mostly involves ‘shouting contests’ with difficult to decipher influences on fitness and survival probability of ‘winning’ and ‘losing’ groups [26]. In some primates, in contrast, intergroup conflict also often occurs within an individual's lifespan yet can be (sub)lethal and comes with significant loss of life and (access to) territories, shelter and mating opportunities [18,168]. Here, recurrent intergroup conflict more likely exerts significant effects on the course of behavioural evolution of a species. Of note is that for some species, different rates and impacts of intergroup conflict are observed in some ecological niches than in others. For example, members of some chimpanzee groups are more often engaged in intergroup fights than members of other chimpanzee groups [16].

Provided intergroup conflict is both frequent and impactful, ultimate consequences may be seen at the level of individuals. Following the logic of natural selection, at least in some species, the physical and mental capacities of successful fighters reproduce and spread more than those of less successful fighters [80,83,138,167]. For example, Massaro *et al.* [44] used 30 years of longitudinal data of 23 male chimpanzees of the Kasekela community, Gombe National Park, in Tanzania, to examine what best explain individual participation in patrol, such as reproductive success, fighting ability and motivation. They found that mating success, as well as fighting ability, were the best correlates of boundary and periphery patrol. Assuming that propensity and capacity for aggressing outsiders is to some degree heritable, these findings suggest that recurrent intergroup conflict may result in groups being increasingly populated with individuals eager and able to fight.

An intriguing possibility is that recurrent intergroup conflict can have ultimate consequences at the group level, especially in terms of how groups are socially organized [172]. One idea is that because groups with more cooperators among their ranks are more likely to win a conflict compared to groups having fewer cooperators [75], not only individual but also group-level mechanisms for within-group cooperation and coordination may survive and spread more than those producing free-riders [173–179]. There is some evidence indeed that, at least in humans, intergroup conflict and warfare reinforce and replace social institutions for cooperation and coordination, including rule of law, religions and social norms (*viz.* cultural evolution [54,180–182]). These patterns may generalize to other species as well. Using evolutionary modelling, Mullon & Lehmann [61] show, for example, how different traits for task specialization (producers and fighters; see §3*b*) can coexist and coevolve within groups in the context of recurrent intergroup conflict. Also, grounded in field observations of intergroup conflicts in banded mongoose, Sankey *et al*. [24] provide evolutionary agent-based modelling of how intergroup competition contributes to the emergence of ‘exploitative’ leaders.

## 5. The scope of the present theme issue

Within this broad framework on the mechanisms and consequences of intergroup conflict across taxa, the contributions to this theme issue make a range of innovations and set goalposts for future research. We list three that crosscut throughout the theme issue.

### (a) Parochial pro-sociality

Studies in psychology and sociology on human warfare and intergroup conflict have traditionally focused on intergroup perceptions, prejudices and histories of conflict as core triggers of violence between groups of people (for reviews, see [74,103,183]). Next to intergroup histories and perceptions, several contributions to the current theme issue reveal parochial pro-sociality and within-group cooperation as key to the initiation and escalation of intergroup conflict. Triki *et al*. [40] review evidence from various social vertebrates that the release of oxytocin strengthens social bonds among group members and that the ensuing parochial pro-sociality motivates participation in intergroup conflict. Likewise, Lemoine *et al*. [18] review evidence that collective grooming and food sharing in chimpanzees prior to intergroup encounters can increase social ties among group members, and stronger social ties enable collective action during violent intergroup encounters. Finally, De Dreu *et al*. [45] show how solidarity among group members and increased parochialism enable costly investment in out-group aggression, forcing out-groups to invest resources in group defence.

Common across these studies is that intergroup conflict resides in enhanced care among individuals for their in-group, which can be fruitfully captured in economic models of social preferences reviewed by Rusch [51]. These studies also suggest that prejudices and negative feelings towards out-groups often observed in humans are a consequence rather than a cause of intergroup conflict. Dogan *et al*. [47] examined this in three natural groups in Ethiopia that vary in how hostile intergroup relations are. Their experiments show that in-group bias largely manifests as positive concern for in-group members combined with no concern for out-group members. Enmity had no effect on in-group bias. These results thus suggest that policy for reducing conflict and its waste may be more effective when (also) focusing on parochial pro-sociality, rather than exclusively focusing on ‘undoing’ negative perceptions of out-groups.

### (b) Within-group heterogeneities and social organization

Past research and theory on intergroup conflict often assumed that groups are composed of highly similar if not identical members. Several contributions relax this simplifying assumption and take seriously that individuals within groups differ in sex, behavioural predispositions, abilities to contribute to collective action and in the fitness costs and benefits they incur from participating in intergroup conflict. As a case in point, Glowacki & McDermott [138] show how in various group-living species, ‘impact individuals’ are pivotal in forming coalitions (*viz*. horizontal task specialization; §3*b*) and in initiating and escalating intergroup conflict. Moreover, these ‘impact’ individuals may benefit disproportionately from group conflict, face a low risk of injury or stand out in terms of boldness of character. Massaro *et al*. [44] suggest, furthermore, how such within-group heterogeneity can emerge and how it is linked to participation in boundary and periphery patrols in male chimpanzees of the Kasekela community. This in turn may shape group-specific temperaments alongside the likelihood of violent intergroup encounters and conflict.

Several contributions draw attention to within-group heterogeneities in horizontal and vertical task specializations during intergroup conflict. Bshary *et al.* [43] examine the possibility that female group members ‘incentivize’ males to engage in intergroup fighting with mating opportunities. Smith *et al.* [17] provide an extensive comparative phylogenetic analysis comprising 72 group-living mammalian species showing that the mammalian modal pattern for participation in intergroup conflict is male-biased, while it is female-biased for collective movements (e.g. migrations, food gathering). Intriguingly, they also find that male-biased participation in intergroup conflicts decreases, and female-biased participation increases with female-biased leadership in movements. Thus, female-biased participation in intergroup conflict only emerged in species with female-biased leadership in collective movement, such as in spotted hyaenas and some lemurs. Smith *et al*. [17] attribute these patterns to costs and benefits of participating in collective movements (e.g. towards food, water, safety) and intergroup conflict (e.g. access to mates or resources).

To some extent, within-group heterogeneities impact, and are further shaped by, partner choice and alliance formation for and during intergroup conflict. Mathew [46] provides extensive ethnographic evidence for the factors underlying partner choice and alliance formation for intergroup raids and warfare among the Turkana. Ridley *et al*. [27] document an intriguing and hitherto poorly understood form of partner choice and alliance formation in pied babblers (*Turdoides bicolor*): kidnapping young from rivalling groups and raising them as one's own. They reason that although raising kidnapped young requires energetic investment and abductees are usually unrelated to their kidnappers, kidnapping may be beneficial in species where group size is critically a limiting factor on territory defence or reproductive fitness, for instance. In groups of pied babblers, they observe kidnapping to be a highly predictable event, especially in groups that fail to raise their own young and that recruitment of young is a critical factor in maintaining territory size.

In theory, recurrent intergroup conflict selects on group-level mechanisms for coordinating collective action (see §4*b*). Several contributions to the current theme issue advance these possibilities with evolutionary agent-based simulations and computational modelling. Bshary *et al.* [43] show how intergroup conflict may be pivotal in creating and maintaining female-dominated multi-male groups. Mullon & Lehmann [61] show how intergroup aggression can be a potent mechanism in favouring within-group social diversity where some group members participate exclusively in group defence and appropriation (scrounger hawks) and others participate only in common pool resource production (producer doves). Finally, with an ecological model and agent-based simulations, Sankey *et al.* [24] show how different forms of leadership can survive in and emerge from intergroup conflict. Their analysis distinguishes ‘exploitative’ leaders who initiate intergroup conflict and monopolize the benefits at little cost to themselves and ‘heroic’ leaders who willingly pay increased costs of conflict participation above and beyond their share of the spoils. Sankey *et al*. find that small group size, low migration rate and frequent interaction between groups increase intergroup competition and the evolution of ‘exploitative’ leadership, while converse patterns favour the emergence of ‘heroic’ leaders and more peaceful outcomes.

### (c) Ecological shifts

The current emphasis on intergroup conflict should not be taken as if groups within structured populations are bound to compete and fight. For example, in bonobos, intergroup encounters can involve some scrimmages but hardly if ever turn violent [18,26]. For many other species, groups often coexist peacefully and cooperate across group boundaries for prolonged periods [49,184]. For example, neighbouring pods of orcas (*Orcinus orca*) in the northern Gulf of Alaska move sequentially through the habitat during periods of relative prey scarcity, thereby effectively limiting intergroup resource competition and avoiding intergroup conflict [185]. There is also evidence that orcas prefer mating with rather than fighting groups of conspecifics upon encountering them [186]. Thus, both within and across species, intergroup conflicts differ in intensity and in frequency (also figure 3), and this requires theory about the rise and fall of intergroup conflict—when and how do peaceful intergroup relations turn violent, and what transitions intergroup conflict into peaceful coexistence and cross-group cooperation?

Rodrigues *et al.* [33] make an important advance in this respect by modelling how social insects reduce the costs of conflict through individual or colony level avoidance, ritualistic behaviours and even group fusion. They also provide a mathematical model of multi-level population structures wherein the increased likelihood of cooperative partners being kin is balanced by increased kin competition, such that neither cooperation nor conflict is favoured. The model by Rodrigues *et al*. [33] provides a useful baseline context in which other intra- and intergroup processes act, tipping the balance towards or away from conflict. One such factor may be changes in the groups' ecology, as suggested by various contributions to the current theme. For instance, Neumann & Pinter-Wolman [32] highlight that groups may need to trade off which resources to compete for under restricted environments with limited resources. In an experimental set-up, both the invasive Argentine ant (*Linepithema humile*) and the native odorous ant (*Tapinoma sessile*) prefer to occupy shelter and food locations. However, when both resources became scarce, the invasive ants controlled the shelter location through aggressive displacement but lost control over food. Several contributions [41,42,45] provide evidence that, for primate groups including humans, ecological shifts through degrading habitats and shrinking territories are among the key factors that transition peaceful coexistence among groups into violent interactions and conflict. These and related findings reveal the potential for climate change alongside extreme weather events as a potentially disruptive force in structured populations across taxa and species.

## 6. Coda

Intergroup conflict can emerge when individuals are nested in groups that share access to scarce resources with adjacent groups of conspecifics. Alone and in combination, the contributions to this theme issue reveal how groups initiate and escalate intergroup conflict and the ultimate consequences in terms of species survival, fitness and social organization. These and related insights together suggest an answer to a question we have not yet asked: how do individuals become organized in groups, allowing for the ‘us versus them’ dynamics that are core to this theme issue? One possible answer from the collection of articles in this theme issue is that population structures emerge and change because individuals form alliances to compete collectively for resources held or demanded by outsider conspecifics. Seen as such, intergroup conflict is not only an intricate part of group-living across species and taxa but also a pivotal mechanism that produces group-living in the first place.

## Data Availability

This article has no additional data.

## Footnotes

1 The terms ‘intergroup conflict’ and ‘warfare’ are sometimes used interchangeably and sometimes used to denote different things. In the present treatise, warfare refers to activities undertaken by a group to settle intergroup conflict in their favour. In theory, warfare can be observed in any species involved in intergroup conflict, and intergroup conflict may or may not involve warfare. Warfare can, but does not have to, take the form of planned and organized violence of one group against another.

2 The current focus on cross-species similarities in the mechanisms and consequences of intergroup conflict should not be taken as if important differences do not exist. For example, humans have comparatively advanced ability for communication and cultural cognition that enable them to initiate, organize and execute intergroup conflict in ways few other species can (further see [53–58]).

## References

[1] Louwe Kooijmans LP. 1993 An early/middle Bronze Age multiple burial at Wassenaar, the Netherlands. *Analecta Praehist. Leiden. 26 End Our Third Decade Pap. Writ. Occas. 30th Anniv. Institutte Prehistory Vol. II* **26**, 1–20.

[2] Golitko M, Keeley LH. 2007 Beating ploughshares back into swords: warfare in the Linearbandkeramik. Antiquity **81**, 332-342. (10.1017/S0003598X00095211)

[3] Jantzen D et al. 2011 A Bronze Age battlefield? weapons and trauma in the Tollense Valley, north-eastern Germany. Antiquity **85**, 417-433. (10.1017/S0003598X00067843)

[4] Fokkens H, Fontijn D. 2013 The Bronze Age in the low countries. In The Oxford handbook of the European bronze age (eds H Fokkens, A Harding), pp. 550-570. Oxford, UK: Oxford University Press.

[5] Keeley L. 1996 War before civilization. The myth of the peaceful savage. Oxford, UK: Oxford University Press.

[6] Kelly RC. 2005 The evolution of lethal intergroup violence. Proc. Natl Acad. Sci. USA **102**, 15 294-15 298. (10.1073/pnas.0505955102) 16129826 PMC1266108

[7] Bowles S. 2009 Did warfare among ancestral hunter-gatherers affect the evolution of human social behaviors? Science **324**, 1293-1298. (10.1126/science.1168112) 19498163

[8] Zefferman MR, Mathew S. 2015 An evolutionary theory of large-scale human warfare: group-structured cultural selection. Evol. Anthr. Issues News Rev. **24**, 50-61. (10.1002/evan.21439) 25914359

[9] Gat A. 2019 Is war in our nature? Hum. Nat. **30**, 149-154. (10.1007/s12110-019-09342-8) 30848430

[10] Kissel M, Kim NC. 2019 The emergence of human warfare: current perspectives. Am. J. Phys. Anthr. **168**, 141-163. (10.1002/ajpa.23751) 30575025

[11] Glowacki L, Wilson ML, Wrangham RW. 2020 The evolutionary anthropology of war. J. Econ. Behav. Organ. **178**, 963-982. (10.1016/j.jebo.2017.09.014)

[12] Fry DP, Söderberg P. 2013 Lethal aggression in mobile forager bands and implications for the origins of war. Science **341**, 270-273. (10.1126/science.1235675) 23869015

[13] Manson JH et al. 1991 Intergroup aggression in chimpanzees and humans [and comments and replies]. Curr. Anthropol. **32**, 369-390. (10.1086/203974)

[14] Gros-Louis J, Perry S, Manson JH. 2003 Violent coalitionary attacks and intraspecific killing in wild white-faced capuchin monkeys (*Cebus capucinus*). Primates **44**, 341-346. (10.1007/s10329-003-0050-z) 12910384

[15] Gilby IC, Brent LJ, Wroblewski EE, Rudicell RS, Hahn BH, Goodall J, Pusey AE. 2013 Fitness benefits of coalitionary aggression in male chimpanzees. Behav. Ecol. Sociobiol. **67**, 373-381. (10.1007/s00265-012-1457-6) 23459197 PMC3582680

[16] Wilson ML et al. 2014 Lethal aggression in *Pan* is better explained by adaptive strategies than human impacts. Nature **513**, 414-417. (10.1038/nature13727) 25230664

[17] Smith JE, Fichtel C, Holmes RK, Kappeler PM, van Vugt M, Jaeggi AV. 2022 Sex bias in intergroup conflict and collective movements among social mammals: male warriors and female guides. Phil. Trans. R. Soc. B **377**, 20210142. (10.1098/rstb.2021.0142) 35369756 PMC8977663

[18] Lemoine SRT, Samuni L, Crockford C, Wittig RM. 2022 Parochial cooperation in wild chimpanzees: a model to explain the evolution of parochial altruism. Phil. Trans. R. Soc. B **377**, 20210149. (10.1098/rstb.2021.0149) 35369746 PMC8977654

[19] Aureli F, Schaffner CM, Verpooten J, Slater K, Ramos-Fernandez G. 2006 Raiding parties of male spider monkeys: insights into human warfare? Am. J. Phys. Anthr. Off. Publ. Am. Assoc. Phys. Anthr. **131**, 486-497. (10.1002/ajpa.20451) 16685723

[20] Benson-Amram S, Heinen VK, Dryer SL, Holekamp KE. 2011 Numerical assessment and individual call discrimination by wild spotted hyaenas, *Crocuta crocuta*. Anim. Behav. **82**, 743-752. (10.1016/j.anbehav.2011.07.004)

[21] Cassidy KA, MacNulty DR, Stahler DR, Smith DW, Mech LD. 2015 Group composition effects on aggressive interpack interactions of gray wolves in Yellowstone National Park. Behav. Ecol. **26**, 1352-1360. (10.1093/beheco/arv081)

[22] Jordan NR, Cherry MI, Manser MB. 2007 Latrine distribution and patterns of use by wild meerkats: implications for territory and mate defence. Anim. Behav. **73**, 613-622. (10.1016/j.anbehav.2006.06.010)

[23] Green PA, Preston EFR, Nicholl MH, Croft DP, Thompson FJ, Cant MA. 2021 Collective defence and behavioural homogeneity during simulated territorial intrusions in banded mongooses (*Mungos mungo*). Ethology **127**, 886-896. (10.1111/eth.13204)

[24] Sankey DWE, Hunt KL, Croft DP, Franks DW, Green PA, Thompson FJ, Johnstone RA, Cant MA. 2022 Leaders of war: modelling the evolution of conflict among heterogeneous groups. Phil. Trans. R. Soc. B **377**, 20210140. (10.1098/rstb.2021.0140) 35369752 PMC8977670

[25] Radford AN, Majolo B, Aureli F. 2016 Within-group behavioural consequences of between-group conflict: a prospective review. Proc. R. Soc. B **283**, 20161567. (10.1098/rspb.2016.1567) PMC5136580 27903869

[26] Morris-Drake A, Kennedy P, Braga Goncalves I, Radford AN. 2022 Variation between species, populations, groups and individuals in the fitness consequences of outgroup conflict. Phil. Trans. R. Soc. B **377**, 20210148. (10.1098/rstb.2021.0148) 35369741 PMC8977661

[27] Ridley AR, Nelson-Flower MJ, Wiley EM, Humphries DJ, Kokko H. 2022 Kidnapping intergroup young: an alternative strategy to maintain group size in the group-living pied babbler (*Turdoides bicolor*). Phil. Trans. R. Soc. B **377**, 20210153. (10.1098/rstb.2021.0153) 35369755 PMC8977656

[28] Bruintjes R, Lynton-Jenkins J, Jones JW, Radford AN. 2016 Out-group threat promotes within-group affiliation in a cooperative fish. Am. Nat. **187**, 274-282. (10.1086/684411) 26807753

[29] Langen TA, Tripet F, Nonacs P. 2000 The red and the black: habituation and the dear-enemy phenomenon in two desert Pheidole ants. Behav. Ecol. Sociobiol. **48**, 285-292. (10.1007/s002650000223)

[30] Reeve HK, Hölldobler B. 2007 The emergence of a superorganism through intergroup competition. Proc. Natl Acad. Sci. USA **104**, 9736-9740. (10.1073/pnas.0703466104) 17517608 PMC1887545

[31] Cunningham JP, Hereward JP, Heard TA, De Barro PJ, West SA. 2014 Bees at war: interspecific battles and nest usurpation in stingless bees. Am. Nat. **184**, 777-786. (10.1086/678399) 25438177

[32] Neumann K, Pinter-Wollman N. 2022 The effect of resource availability on interspecific competition between a native and an invasive ant. Phil. Trans. R. Soc. B **377**, 20210146. (10.1098/rstb.2021.0146) 35369748 PMC8977667

[33] Rodrigues AMM, Barker JL, Robinson EJH. 2022 From inter-group conflict to inter-group cooperation: insights from social insects. Phil. Trans. R. Soc. B **377**, 20210466. (10.1098/rstb.2021.0466) 35369743 PMC8977659

[34] Leitenberg M. 2006 Deaths in wars and conflicts in the 20th century. Ithaca, NY: Cornell University, Peace Studies Program.

[35] Harbom L, Melander E, Wallensteen P. 2008 Dyadic dimensions of armed conflict, 1946—2007. J. Peace Res. **45**, 697-710. (10.1177/0022343308094331)

[36] Mabelis AA. 1978 Wood ant wars the relationship between aggression and predation in the red wood ant (*Formica polyctena* forst.). Neth. J. Zool. **29**, 451-620. (10.1163/002829679X00016)

[37] Fransen S, Ruiz I, Vargas-Silva C. 2017 Return migration and economic outcomes in the conflict context. World Dev. **95**, 196-210. (10.1016/j.worlddev.2017.02.015)

[38] Affek AN, Wolski J, Zachwatowicz M, Ostafin K, Radeloff VC. 2021 Effects of post-WWII forced displacements on long-term landscape dynamics in the Polish Carpathians. Landsc. Urban Plan. **214**, 104164. (10.1016/j.landurbplan.2021.104164)

[39] Bendavid E et al. 2021 The effects of armed conflict on the health of women and children. Lancet **397**, 522-532. (10.1016/S0140-6736(21)00131-8) 33503456 PMC7612212

[40] Triki Z, Daughters K, De Dreu CKW. 2022 Oxytocin has ‘tend-and-defend’ functionality in group conflict across social vertebrates. Phil. Trans. R. Soc. B **377**, 20210137. (10.1098/rstb.2021.0137) 35369742 PMC8977669

[41] García MG, de Guinea M, Bshary R, van de Waal E. 2022 Drivers and outcomes of between-group conflict in vervet monkeys. Phil. Trans. R. Soc. B **377**, 20210145. (10.1098/rstb.2021.0145) 35369750 PMC8977665

[42] Brown M, Steinitz R, Emery Thompson E. 2022 Wins and losses in intergroup conflicts reflect energy balance in red-tailed monkeys. Phil. Trans. R. Soc. B **377**, 20210152. (10.1098/rstb.2021.0152) 35369757 PMC8977655

[43] Bshary R, Richter X-YL, van Schaik C. 2022 Male services during between-group conflict: the ‘hired gun’ hypothesis revisited. Phil. Trans. R. Soc. B **377**, 20210150. (10.1098/rstb.2021.0150) 35369754 PMC8977666

[44] Massaro A, Gilby IC, Desai N, Weiss A, Feldblum JT, Pusey AE, Wilson ML. 2022 Correlates of individual participation in boundary patrols by male chimpanzees. Phil. Trans. R. Soc. B **377**, 20210151. (10.1098/rstb.2021.0151) 35369753 PMC8977668

[45] De Dreu CKW, Gross J, Reddmann L. 2022 Environmental stress increases out-group aggression and intergroup conflict in humans. Phil. Trans. R. Soc. B **377**, 20210147. (10.1098/rstb.2021.0147) 35369744 PMC8977653

[46] Mathew S. 2021 Turkana warriors' call to arms: how an egalitarian society mobilizes for cattle raids. Phil. Trans. R. Soc. B **377**, 20210144. (10.1098/rstb.2021.0144) PMC8977660 35369747

[47] Doğan G, Glowacki L, Rusch H. 2022 Are strangers just enemies you have not yet met? Group homogeneity, not intergroup relations, shapes ingroup bias in three natural groups. Phil. Trans. R. Soc. B **377**, 20210419. (10.1098/rstb.2021.0419) 35369759 PMC8977658

[48] Bornstein G. 2003 Intergroup conflict: individual, group, and collective interests. Pers. Soc. Psychol. Rev. Off. J. Soc. Pers. Soc. Psychol. Inc **7**, 129-145. (10.1207/S15327957PSPR0702_129-145) 12676644

[49] De Dreu CKW, Gross J, Fariña A, Ma Y. 2020 Group cooperation, carrying-capacity stress, and intergroup conflict. Trends Cogn. Sci. **24**, 760-776. 32620334 10.1016/j.tics.2020.06.005

[50] Rusch H, Gavrilets S. 2020 The logic of animal intergroup conflict: a review. J. Econ. Behav. Organ. **178**, 1014-1030. (10.1016/j.jebo.2017.05.004)

[51] Rusch H. 2022 Modelling behaviour in intergroup conflicts: a review of microeconomic approaches. Phil. Trans. R. Soc. B **377**, 20210135. (10.1098/rstb.2021.0135) 35369749 PMC8977652

[52] Wrangham RW. 1999 Evolution of coalitionary killing. Am. J. Phys. Anthr. **110**, 1-30. (10.1002/(SICI)1096-8644(1999)110:29+<1::AID-AJPA2>3.0.CO;2-E) 10601982

[53] De Dreu CKW, Gross J. 2019 Revisiting the form and function of conflict: neurobiological, psychological, and cultural mechanisms for attack and defense within and between groups. Behav. Brain Sci. **42**, e116. (10.1017/S0140525X18002170) 30251617

[54] Richerson P et al. 2016 Cultural group selection plays an essential role in explaining human cooperation: a sketch of the evidence. Behav. Brain Sci. **39**, e30. (10.1017/S0140525X1400106X) 25347943

[55] Moffett MW. 2013 Human identity and the volution of societies. Hum. Nat. 24, 219-267. 23813244 10.1007/s12110-013-9170-3

[56] Shettleworth SJ, 2012 Modularity, comparative cognition and human uniqueness. Phil. trans. R. Soc. B 367, 2794-2802. (10.1098/rstb.2012.0211) 22927578 PMC3427548

[57] Tomasello M, Carpenter M, Call J, Behne T, Moll H. 2005 Understanding and sharing intentions: the origins of cultural cognition. Behav. Brain Sci. 28, 675-691. 16262930 10.1017/S0140525X05000129

[58] Voorhees B, Read D, Gabora L. 2020 Identity, kinship, and the evolution of cooperation. Curr. Anthropol. 61, 194-218.

[59] Kappeler PM. 2019 A framework for studying social complexity. Behav. Ecol. Sociobiol. **73**, 13. (10.1007/s00265-018-2601-8)

[60] Wright S. 1931 Evolution in Mendelian populations. Genetics **16**, 97. (10.1093/genetics/16.2.97) 17246615 PMC1201091

[61] Mullon C, Lehmann L. 2018 Eco-evolutionary dynamics in metacommunities: ecological inheritance, helping within species, and harming between species. Am. Nat. **192**, 664-686. (10.1086/700094) 30444662

[62] Mullon C, Wakano JY, Ohtsuki H. 2021 Coevolutionary dynamics of genetic traits and their long-term extended effects under non-random interactions. J. Theor. Biol. **525**, 110750. (10.1016/j.jtbi.2021.110750) 33957155

[63] Mullon C, Lehmann L. 2022 Evolution of warfare by resource raiding favours polymorphism in belligerence and bravery. Phil. Trans. R. Soc. B **377**, 20210136. (10.1098/rstb.2021.0136) 35369745 PMC8977657

[64] Rapoport A, Anatol R. 1960 Fights, games, and debates. Ann Arbor, MI: University of Michigan Press.

[65] De Dreu CK, Pliskin R, Rojek-Giffin M, Méder Z, Gross J. 2021 Political games of attack and defence. Phil. Trans. R. Soc. B **376**, 20200135. (10.1098/rstb.2020.0135) 33611990 PMC7934902

[66] Durham WH. 1976 Resource competition and human aggression, part I: a review of primitive war. Q. Rev. Biol. **51**, 385-415. (10.1086/409471)

[67] Rusch H. 2014 The two sides of warfare. Hum. Nat. **25**, 359-377. (10.1007/s12110-014-9199-y) 24928285

[68] Glowacki L, Isakov A, Wrangham RW, McDermott R, Fowler JH, Christakis NA. 2016 Formation of raiding parties for intergroup violence is mediated by social network structure. Proc. Natl Acad. Sci. USA **113**, 12 114-12 119. (10.1073/pnas.1610961113) 27790996 PMC5086992

[69] Gavrilets S, Fortunato L. 2014 A solution to the collective action problem in between-group conflict with within-group inequality. Nat. Commun. **5**, 1-11. (10.1038/ncomms4526) PMC3974216 24667443

[70] Schelling TC. 1980 The strategy of conflict. Cambridge, MA: Harvard University Press.

[71] Duguid S, Melis AP. 2020 How animals collaborate: underlying proximate mechanisms. Wiley Interdiscip. Rev. Cogn. Sci. **11**, e1529. (10.1002/wcs.1529) 32342659

[72] van Dijk E, De Dreu CKW. 2021 Experimental games and social decision making. Annu. Rev. Psychol. **72**, 415-438. (10.1146/annurev-psych-081420-110718) 33006926

[73] De Dreu CKW, Gross J, Méder Z, Giffin M, Prochazkova E, Krikeb J, Columbus S. 2016 In-group defense, out-group aggression, and coordination failures in intergroup conflict. Proc. Natl Acad. Sci. USA **113**, 10 524-10 529. (10.1073/pnas.1605115113) 27601640 PMC5035908

[74] Majolo B, deBortoli Vizioli A, Martínez-Íñigo L, Lehmann J. 2020 Effect of group size and individual characteristics on intergroup encounters in primates. Int. J. Primatol. **41**, 325-341. (10.1007/s10764-019-00119-5)

[75] Wilson DS, Wilson EO. 2007 Rethinking the theoretical foundation of sociobiology. Q. Rev. Biol. **82**, 327-348. (10.1086/522809) 18217526

[76] Vermeij GJ. 1982 Unsuccessful predation and evolution. Am. Nat. **120**, 701-720. (10.1086/284025)

[77] Koch F, Signer J, Kappeler PM, Fichtel C. 2016 The role of the residence-effect on the outcome of intergroup encounters in Verreaux's sifakas. Sci. Rep. **6**, 1-7. (10.1038/srep28457) 27328940 PMC4916469

[78] Rubin JZ, Pruitt DG, Kim SH. 1994 Social conflict: escalation, stalemate, and settlement. New York, NY: Mcgraw-Hill Book Company.

[79] De Dreu CK. 2010 Social conflict: the emergence and consequences of struggle and negotiation. Handb. Soc. Psychol. 2, 983-1023.

[80] Dawkins R, Krebs JR. 1979 Arms races between and within species. Proc. R. Soc. Lond. B **205**, 489-511. (10.1098/rspb.1979.0081) 42057

[81] Vermeij GJ. 1994 The evolutionary interaction among species: selection, escalation, and coevolution. Annu. Rev. Ecol. Syst. **25**, 219-236. (10.1146/annurev.es.25.110194.001251)

[82] Hembry DH, Weber MG. 2020 Ecological interactions and macroevolution: a new field with old roots. Annu. Rev. Ecol. Evol. Syst. **51**, 215-243. (10.1146/annurev-ecolsys-011720-121505)

[83] Smith J, Price GR. 1973 The logic of animal conflict. Nature **246**, 15-18. (10.1038/246015a0)

[84] Bateson P, Laland KN. 2013 Tinbergen's four questions: an appreciation and an update. Trends Ecol. Evol. **28**, 712-718. (10.1016/j.tree.2013.09.013) 24144467

[85] Tinbergen N. 1963 On aims and methods of ethology. Z. Tierpsychol. **20**, 410-433. (10.1111/j.1439-0310.1963.tb01161.x)

[86] Doğan G, Glowacki L, Rusch H. 2018 Spoils division rules shape aggression between natural groups. Nat. Hum. Behav. **2**, 322-326. (10.1038/s41562-018-0338-z) 30962600

[87] Johnstone RA, Cant MA, Cram D, Thompson FJ. 2020 Exploitative leaders incite intergroup warfare in a social mammal. Proc. Natl Acad. Sci. USA **117**, 29 759-29 766. (10.1073/pnas.2003745117) 33168743 PMC7703641

[88] Gintis H, Smith EA, Bowles S. 2001 Costly signaling and cooperation. J. Theor. Biol. **213**, 103-119. (10.1006/jtbi.2001.2406) 11708857

[89] Przepiorka W, Diekmann A. 2021 Parochial cooperation and the emergence of signalling norms. Phil. Trans. R. Soc. B **376**, 20200294. (10.1098/rstb.2020.0294) 34601914 PMC8487749

[90] Romano A, Giardini F, Columbus S, de Kwaadsteniet EW, Kisfalusi D, Triki Z, Snijders C, Hagel K. 2021 Reputation and socio-ecology in humans. Phil. Trans. R. Soc. B **376**, 20200295. (10.1098/rstb.2020.0295) 34601915 PMC8487743

[91] Hamilton WD. 1964 The genetical evolution of social behaviour. II. J. Theor. Biol. **7**, 17-52. (10.1016/0022-5193(64)90039-6) 5875340

[92] De Dreu CK, Weingart LR, Kwon S. 2000 Influence of social motives on integrative negotiation: a meta-analytic review and test of two theories. J. Pers. Soc. Psychol. **78**, 889. (10.1037/0022-3514.78.5.889) 10821196

[93] Yamagishi T, Kiyonari T. 2000 The group as the container of generalized reciprocity. Soc. Psychol. Q. **63**, 116-132. (10.2307/2695887)

[94] Silk JB. 2007 The adaptive value of sociality in mammalian groups. Phil. Trans. R. Soc. B **362**, 539-559. (10.1098/rstb.2006.1994) 17363359 PMC2346516

[95] Tokuyama N, Furuichi T. 2016 Do friends help each other? patterns of female coalition formation in wild bonobos at Wamba. Anim. Behav. **119**, 27-35. (10.1016/j.anbehav.2016.06.021)

[96] Balliet D, Wu J, De Dreu CKW. 2014 Ingroup favoritism in cooperation: a meta-analysis. Psychol. Bull. **140**, 1556-1581. (10.1037/a0037737) 25222635

[97] Arseneau TJM, Taucher A-L, van Schaik CP, Willems EP. 2015 Male monkeys fight in between-group conflicts as protective parents and reluctant recruits. Anim. Behav. **110**, 39-50. (10.1016/j.anbehav.2015.09.006)

[98] De Dreu CK, Fariña A, Gross J, Romano A. 2022 Pro-sociality as a foundation for intergroup conflict. Curr. Opin. Psychol. **44**, 112-116. (10.1016/j.copsyc.2021.09.002) 34610546

[99] Romano A, Sutter M, Liu JH, Balliet D. 2021 Political ideology, cooperation and national parochialism across 42 nations. Phil. Trans. R. Soc. B **376**, 20200146. (10.1098/rstb.2020.0146) 33611989 PMC7934968

[100] Cheng L, Lucchesi S, Mundry R, Samuni L, Deschner T, Surbeck M. 2021 Variation in aggression rates and urinary cortisol levels indicates intergroup competition in wild bonobos. Horm. Behav. **128**, 104914. (10.1016/j.yhbeh.2020.104914) 33373622

[101] Buchan NR, Grimalda G, Wilson R, Brewer M, Fatas E, Foddy M. 2009 Globalization and human cooperation. Proc. Natl Acad. Sci. USA **106**, 4138-4142. (10.1073/pnas.0809522106) 19255433 PMC2657440

[102] Aaldering H, Ten Velden FS, van Kleef GA, De Dreu CK. 2018 Parochial cooperation in nested intergroup dilemmas is reduced when it harms out-groups. J. Pers. Soc. Psychol. **114**, 909. (10.1037/pspi0000125) 29389154

[103] Pisor AC, Surbeck M. 2019 The evolution of intergroup tolerance in nonhuman primates and humans. Evol. Anthr. Issues News Rev. **28**, 210-223. (10.1002/evan.21793) 31386248

[104] Foster KR, Wenseleers T, Ratnieks FL. 2001 Spite: Hamilton's unproven theory. Annales zoologici fennici 38, 229-238.

[105] Halevy N, Bornstein G, Sagiv L. 2008 ‘In-group love’ and ‘out-group hate’ as motives for individual participation in intergroup conflict: a new game paradigm. Psychol. Sci. **19**, 405-411. (10.1111/j.1467-9280.2008.02100.x) 18399895

[106] Smead R, Forber P. 2013 The evolutionary dynamics of spite in finite populations. Evol. Int. J. Org. Evol. **67**, 698-707. (10.1111/j.1558-5646.2012.01831.x) 23461321

[107] De Dreu CKW, Greer LL, Handgraaf MJJ, Shalvi S, Van Kleef GA, Baas M, Ten Velden FS, Van Dijk E, Feith SWW. 2010 The neuropeptide oxytocin regulates parochial altruism in intergroup conflict among humans. Science **328**, 1408-1411. (10.1126/science.1189047) 20538951

[108] Riek BM, Mania EW, Gaertner SL. 2006 Intergroup threat and outgroup attitudes: a meta-analytic review. Pers. Soc. Psychol. Rev. **10**, 336-353. (10.1207/s15327957pspr1004_4) 17201592

[109] Arnott G, Elwood RW. 2009 Assessment of fighting ability in animal contests. Anim. Behav. **77**, 991-1004. (10.1016/j.anbehav.2009.02.010)

[110] Abbink K, de Haan T. 2014 Trust on the brink of Armageddon: the first-strike game. Eur. Econ. Rev. **67**, 190-196. (10.1016/j.euroecorev.2014.01.009)

[111] Bohn M, Eckert J, Hanus D, Haun DBM. 2021 A longitudinal study of great ape cognition: stability, reliability and the influence of individual characteristics. Proc. Ann. Meet. Cog. Sci. Soc. 43. (10.31234/osf.io/pdt5w)

[112] Koch F, Signer J, Kappeler PM, Fichtel C. 2016 Intergroup encounters in Verreaux's sifakas (*Propithecus verreauxi*): who fights and why? Behav. Ecol. Sociobiol. **70**, 797-808. (10.1007/s00265-016-2105-3) 27194822 PMC4841837

[113] Madden JR, Clutton-Brock TH. 2011 Experimental peripheral administration of oxytocin elevates a suite of cooperative behaviours in a wild social mammal. Proc. Biol. Sci. **278**, 1189-1194. (10.1098/rspb.2010.1675) 20926437 PMC3049071

[114] Halevy N, Chou EY, Cohen TR, Livingston RW. 2012 Status conferral in intergroup social dilemmas: behavioral antecedents and consequences of prestige and dominance. J. Pers. Soc. Psychol. **102**, 351. (10.1037/a0025515) 21928914

[115] Rusch H. 2022 Heroic behavior: A review of the literature on high-stakes altruism in the wild. Curr. Opin. Psychol. **43**, 238-243. (10.1016/j.copsyc.2021.07.024) 34454246

[116] Clutton-Brock TH, Parker GA. 1995 Punishment in animal societies. Nature **373**, 209-216. (10.1038/373209a0) 7816134

[117] Frank SA. 1995 Mutual policing and repression of competition in the evolution of cooperative groups. Nature **377**, 520-522. (10.1038/377520a0) 7566147

[118] Raihani NJ, Grutter AS, Bshary R. 2010 Punishers benefit from third-party punishment in fish. Science **327**, 171. (10.1126/science.1183068) 20056883

[119] Ratnieks FL, Foster KR, Wenseleers T. 2006 Conflict resolution in insect societies. Annu. Rev. Entomol. **51**, 581-608. (10.1146/annurev.ento.51.110104.151003) 16332224

[120] Abbink K, Brandts J, Herrmann B, Orzen H. 2010 Intergroup conflict and intra-group punishment in an experimental contest game. Am. Econ. Rev. **100**, 420-447. (10.1257/aer.100.1.420)

[121] Mathew S, Boyd R. 2011 Punishment sustains large-scale cooperation in prestate warfare. Proc. Natl Acad. Sci. USA **108**, 11 375-11 380. (10.1073/pnas.1105604108) PMC3136302 21670285

[122] Macfarlan SJ, Walker RS, Flinn MV, Chagnon NA. 2014 Lethal coalitionary aggression and long-term alliance formation among Yanomamö men. Proc. Natl Acad. Sci. USA **111**, 16 662-16 669. (10.1073/pnas.1418639111) PMC4250129 25349394

[123] Whitehouse H, McQuinn B, Buhrmester M, Swann WB. 2014 Brothers in arms: Libyan revolutionaries bond like family. Proc. Natl Acad. Sci. USA **111**, 17 783-17 785. (10.1073/pnas.1416284111) PMC4273349 25385591

[124] Yang J, Zhang H, Ni J, De Dreu CKW, Ma Y. 2020 Within-group synchronization in the prefrontal cortex associates with intergroup conflict. Nat. Neurosci. **23**, 754-760. (10.1038/s41593-020-0630-x) 32341541

[125] Demartsev V, Strandburg-Peshkin A, Ruffner M, Manser M. 2018 Vocal turn-taking in meerkat group calling sessions. Curr. Biol. **28**, 3661-3666.e3. (10.1016/j.cub.2018.09.065) 30416063

[126] Leibbrandt A, Sääksvuori L. 2012 Communication in intergroup conflicts. Eur. Econ. Rev. **56**, 1136-1147. (10.1016/j.euroecorev.2012.05.003)

[127] Conradt L, Roper TJ. 2005 Consensus decision making in animals. Trends Ecol. Evol. **20**, 449-456. (10.1016/j.tree.2005.05.008) 16701416

[128] King AJ, Cowlishaw G. 2009 Leaders, followers, and group decision-making. Commun. Integr. Biol. **2**, 147-150. (10.4161/cib.7562) 19513268 PMC2686370

[129] Gächter S, Renner E. 2018 Leaders as role models and ‘belief managers’ in social dilemmas. J. Econ. Behav. Organ. **154**, 321-334. (10.1016/j.jebo.2018.08.001)

[130] Walker RH, King AJ, McNutt JW, Jordan NR. 2017 Sneeze to leave: African wild dogs (*Lycaon pictus*) use variable quorum thresholds facilitated by sneezes in collective decisions. Proc. R. Soc. B **284**, 20170347. (10.1098/rspb.2017.0347) PMC5597819 28878054

[131] Sand H, Wikenros C, Wabakken P, Liberg O. 2006 Effects of hunting group size, snow depth and age on the success of wolves hunting moose. Anim. Behav. **72**, 781-789. (10.1016/j.anbehav.2005.11.030)

[132] Levati MV, Sutter M, Van der Heijden E. 2007 Leading by example in a public goods experiment with heterogeneity and incomplete information. J. Confl. Resolut. **51**, 793-818. (10.1177/0022002707302796)

[133] Glowacki L, von Rueden C. 2015 Leadership solves collective action problems in small-scale societies. Phil. Trans. R. Soc. B **370**, 20150010. (10.1098/rstb.2015.0010) 26503683 PMC4633846

[134] Loerakker B, van Winden F. 2017 Emotional leadership in an intergroup conflict game experiment. J. Econ. Psychol. **63**, 143-167. (10.1016/j.joep.2017.03.009)

[135] Zhang H, Gross J, De Dreu C, Ma Y. 2019 Oxytocin promotes coordinated out-group attack during intergroup conflict in humans. Elife **8**, e40698. (10.7554/eLife.40698) 30681410 PMC6347450

[136] Fjerdingstad EJ, Crozier RH. 2006 The evolution of worker caste diversity in social insects. Am. Nat. **167**, 390-400. (10.1086/499545) 16673347

[137] Kronauer DJ, Libbrecht R. 2018 Back to the roots: the importance of using simple insect societies to understand the molecular basis of complex social life. Curr. Opin. Insect Sci. **28**, 33-39. (10.1016/j.cois.2018.03.009) 30551765

[138] Glowacki L, McDermott R. 2022 Key individuals catalyse intergroup violence. Phil. Trans. R. Soc. B **377**, 20210141. (10.1098/rstb.2021.0141) 35369758 PMC8977664

[139] Melis AP, Hare B, Tomasello M. 2006 Chimpanzees recruit the best collaborators. Science **311**, 1297-1300. (10.1126/science.1123007) 16513985

[140] Prieur J, Pika S. 2020 Gorillas'(*Gorilla g. gorilla*) knowledge of conspecifics’ affordances: intraspecific social tool use for food acquisition. Primates **61**, 583-591. (10.1007/s10329-020-00805-6) 32166437 PMC7347707

[141] Martin JS, Koski SE, Bugnyar T, Jaeggi AV, Massen JJ. 2021 Prosociality, social tolerance and partner choice facilitate mutually beneficial cooperation in common marmosets, *Callithrix jacchus*. Anim. Behav. **173**, 115-136. (10.1016/j.anbehav.2020.12.016)

[142] Torres Ortiz S, Stedt J, Midtiby HS, Egemose HD, Wahlberg M. 2021 Group hunting in harbour porpoises (*Phocoena phocoena*). Can. J. Zool. **99**, 511-520. (10.1139/cjz-2020-0289)

[143] Vail AL, Manica A, Bshary R. 2013 Referential gestures in fish collaborative hunting. Nat. Commun. **4**, 1-7. (10.1038/ncomms2781) 23612306

[144] Lehman C, Loberg S, Wilson M, Gorham E. 2021 Ecology of the Anthropocene signals hope for consciously managing the planetary ecosystem. Proc. Natl Acad. Sci. USA **118**, e2024150118. (10.1073/pnas.2024150118) 34244429 PMC8285894

[145] Read D, LeBlanc S. 2003 Population growth, carrying capacity, and conflict. Curr. Anthropol. **44**, 59-85. (10.1086/344616)

[146] Codding BF, Parker AK, Jones TL. 2019 Territorial behavior among Western North American foragers: allee effects, within group cooperation, and between group conflict. Quat. Int. **518**, 31-40. (10.1016/j.quaint.2017.10.045)

[147] Mwalyosi RB. 1991 Population growth, carrying capacity and sustainable development in south-west Masailand. J. Environ. Manage. **33**, 175-187. (10.1016/S0301-4797(05)80094-5)

[148] Tanaka M. 2007 Habitat use and social structure of a brown lemur hybrid population in the Berenty Reserve, Madagascar. Am. J. Primatol. Off. J. Am. Soc. Primatol. **69**, 1189-1194. (10.1002/ajp.20416 17294429

[149] Madden GD, Karsten JK, Ledogar SH, Schmidt R, Sokhatsky MP. 2018 Violence at Verteba Cave, Ukraine: new insights into the Late Neolithic intergroup conflict. Int. J. Osteoarchaeol. **28**, 44-53. (10.1002/oa.2633)

[150] Tucker MA et al. 2018 Moving in the Anthropocene: global reductions in terrestrial mammalian movements. Science **359**, 466-469. (10.1126/science.aam9712) 29371471

[151] Weitzel EM, Codding BF, Carmody SB, Zeanah DW. 2020 Food production and domestication produced both cooperative and competitive social dynamics in Eastern North America. Environ. Archaeol. 1-14.

[152] Nakagawa T, Tamura K, Yamaguchi Y, Matsumoto N, Matsugi T, Nakao H. 2021 Population pressure and prehistoric violence in the Yayoi period of Japan. J. Archaeol. Sci. **132**, 105420. (10.1016/j.jas.2021.105420)

[153] Smaldino PE, Newson L, Schank JC, Richerson PJ. 2013 Simulating the evolution of the human family: cooperative breeding increases in harsh environments. PLoS ONE **8**, e80753. (10.1371/journal.pone.0080753) 24278318 PMC3835414

[154] Smith JE, Kolowski JM, Graham KE, Dawes SE, Holekamp KE. 2008 Social and ecological determinants of fission–fusion dynamics in the spotted hyaena. Anim. Behav. **76**, 619-636. (10.1016/j.anbehav.2008.05.001)

[155] Burke MB, Miguel E, Satyanath S, Dykema JA, Lobell DB. 2009 Warming increases the risk of civil war in Africa. Proc. Natl Acad. Sci. USA **106**, 20 670-20 674. (10.1073/pnas.0907998106) PMC2781059 19934048

[156] Allen MW, Bettinger RL, Codding BF, Jones TL, Schwitalla AW. 2016 Resource scarcity drives lethal aggression among prehistoric hunter-gatherers in central California. Proc. Natl Acad. Sci. USA **113**, 12 120-12 125. (10.1073/pnas.1607996113) 27790997 PMC5087046

[157] Von Uexkull N, Croicu M, Fjelde H, Buhaug H. 2016 Civil conflict sensitivity to growing-season drought. Proc. Natl Acad. Sci. USA **113**, 12 391-12 396. (10.1073/pnas.1607542113) 27791091 PMC5098672

[158] Thompson FJ, Marshall HH, Vitikainen EI, Cant MA. 2017 Causes and consequences of intergroup conflict in cooperative banded mongooses. Anim. Behav. **126**, 31-40. (10.1016/j.anbehav.2017.01.017)

[159] Hodgkin J, Félix M-A, Clark LC, Stroud D, Gravato-Nobre MJ. 2013 Two leucobacter strains exert complementary virulence on caenorhabditis including death by worm-star formation. Curr. Biol. **23**, 2157-2161. (10.1016/j.cub.2013.08.060) 24206844 PMC3898767

[160] Granato ET, Meiller-Legrand TA, Foster KR. 2019 The evolution and ecology of bacterial warfare. Curr. Biol. **29**, R521-R537. (10.1016/j.cub.2019.04.024) 31163166

[161] Wagner Z, Heft-Neal S, Bhutta ZA, Black RE, Burke M, Bendavid E. 2018 Armed conflict and child mortality in Africa: a geospatial analysis. Lancet **392**, 857-865. (10.1016/S0140-6736(18)31437-5) 30173907 PMC6338336

[162] Bauer M, Blattman C, Chytilová J, Henrich J, Miguel E, Mitts T. 2016 Can war foster cooperation? J. Econ. Perspect. **30**, 249-274. (10.1257/jep.30.3.249)

[163] Radford AN. 2008 Duration and outcome of intergroup conflict influences intragroup affiliative behaviour. Proc. R. Soc. B **275**, 2787-2791. (10.1098/rspb.2008.0787) PMC2605834 18765344

[164] Samuni L, Preis A, Mundry R, Deschner T, Crockford C, Wittig RM. 2017 Oxytocin reactivity during intergroup conflict in wild chimpanzees. Proc. Natl Acad. Sci. USA **114**, 268-273. (10.1073/pnas.1616812114) 28028227 PMC5240673

[165] Kappeler PM, Cremer S, Nunn CL. 2015 Sociality and health: impacts of sociality on disease susceptibility and transmission in animal and human societies. Phil. Trans. R. Soc. B 370, 20140116. (10.1098/rstb.2014.0116) 25870402 PMC4410382

[166] Jaeggi AV, Trumble BC, Brown M. 2018 Group-level competition influences urinary steroid hormones among wild red–tailed monkeys, indicating energetic costs. Am. J. Primatol. **80**, e22757. (10.1002/ajp.22757) 29635811

[167] Alexander RD, Bargia G. 1978 Group selection, altruism, and the levels of organization of life. Annu. Rev. Ecol. Syst. **9**, 449-474. (10.1146/annurev.es.09.110178.002313)

[168] Samuni L, Preis A, Deschner T, Crockford C, Wittig RM. 2018 Reward of labor coordination and hunting success in wild chimpanzees. Commun. Biol. **1**, 1-9. (10.1038/s42003-018-0142-3) 30272017 PMC6131550

[169] Choi J-K, Bowles S. 2007 The coevolution of parochial altruism and war. Science **318**, 636-640. (10.1126/science.1144237) 17962562

[170] Gross J, De Dreu CKW. 2019 The rise and fall of cooperation through reputation and group polarization. Nat. Comm. **10**, 776. (10.1038/s41467-019-08727-8) PMC6377668 30770812

[171] Dyble M. 2021 The evolution of altruism through war is highly sensitive to population structure and to civilian and fighter mortality. Proc. Natl Acad. Sci. USA **118**, e2011142118. (10.1073/pnas.2011142118) 33836563 PMC7980410

[172] Kappeler PM, van Schaik CP. 2002 Evolution of primate social systems. Int. J. Primatol. **23**, 707-740. (10.1023/A:1015520830318)

[173] Darwin C. 1859 The origin of species and The descent of man. New York, NY: The Modern Library.

[174] Plavcan JM, van Schaik CP, Kappeler PM. 1995 Competition, coalitions and canine size in primates. J. Hum. Evol. **28**, 245-276. (10.1006/jhev.1995.1019)

[175] Bowles S, Choi J-K, Hopfensitz A. 2003 The co-evolution of individual behaviors and social institutions. J. Theor. Biol. **223**, 135-147. (10.1016/S0022-5193(03)00060-2) 12814597

[176] Rusch H. 2014 The evolutionary interplay of intergroup conflict and altruism in humans: a review of parochial altruism theory and prospects for its extension. Proc. R. Soc. B **281**, 20141539. (10.1098/rspb.2014.1539) PMC4211448 25253457

[177] Puurtinen M, Heap S, Mappes T. 2015 The joint emergence of group competition and within-group cooperation. Evol. Hum. Behav. **36**, 211-217. (10.1016/j.evolhumbehav.2014.11.005)

[178] Hare B. 2017 Survival of the friendliest: homo sapiens evolved via selection for prosociality. Annu. Rev. Psychol. **68**, 155-186. (10.1146/annurev-psych-010416-044201) 27732802

[179] Micheletti AJ, Ruxton GD, Gardner A. 2017 Intrafamily and intragenomic conflicts in human warfare. Proc. R. Soc. B **284**, 20162699. (10.1098/rspb.2016.2699) PMC5326533 28228515

[180] Soltis J, Boyd R, Richerson PJ. 1995 Can group-functional behaviors evolve by cultural group selection?: An empirical test. Curr. Anthropol. **36**, 473-494. (10.1086/204381)

[181] Turchin P, Currie TE, Turner EA, Gavrilets S. 2013 War, space, and the evolution of old world complex societies. Proc. Natl Acad. Sci. USA **110**, 16 384-16 389. (10.1073/pnas.1308825110) PMC3799307 24062433

[182] Jackson JC, Gelfand M, Ember CR. 2020 A global analysis of cultural tightness in non-industrial societies. Proc. R. Soc. B **287**, 20201036. (10.1098/rspb.2020.1036) PMC7423486 32605518

[183] Haslam N. 2006 Dehumanization: an integrative review. Pers. Soc. Psychol. Rev. 10, 252-264. (10.1207/s15327957pspr1003_4) 16859440

[184] Robinson EJH, Barker JL. 2017 Inter-group cooperation in humans and other animals. Biol. Lett. **13**, 20160793. (10.1098/rsbl.2016.0793) 28250206 PMC5377026

[185] Yurk H, Filatova O, Matkin CO, Barrett-Lennard LG, Brittain M. 2010 Sequential habitat use by two resident killer whale (*Orcinus orca*) clans in Resurrection Bay, Alaska, as determined by remote acoustic monitoring. Aquat. Mamm. **36**, 67-78. (10.1578/AM.36.1.2010.67)

[186] Martien KK, Taylor BL, Chivers SJ, Mahaffy SD, Gorgone AM, Baird RW. 2019 Fidelity to natal social groups and mating within and between social groups in an endangered false killer whale population. Endanger. Species Res. **40**, 219-230. (10.3354/esr00995)

